# How do patients and health care professionals perceive de-implementation of routine follow-ups after total hip or knee arthroplasty? Protocol for a nested qualitative study within a hybrid effectiveness de-implementation trial

**DOI:** 10.1371/journal.pone.0330652

**Published:** 2025-08-28

**Authors:** Lex D. de Jong, Dominique C. Baas, Lidy A.C. Roubos, Jantsje H. Pasma, Ariena J. Rasker, Marijn Rutgers, Ronald A.W. Verhagen, Sigrid N.W. Vorrink, Nienke W. Willigenburg, Rudolf W. Poolman

**Affiliations:** 1 Department of Orthopaedics, Rijnstate Hospital, Arnhem, the Netherlands; 2 Department of Orthopaedic Surgery, Tergooi Medical Center, Hilversum, the Netherlands; 3 Joint Research, Department of Orthopaedic Surgery, OLVG Hospital, Amsterdam, the Netherlands; 4 Department of Orthopaedic Surgery, Leiden University Medical Center, Leiden, the Netherlands; 5 Department Orthopaedic Surgery, Reinier Haga Orthopedic Centre, Zoetermeer, the Netherlands; Public Library of Science, UNITED KINGDOM OF GREAT BRITAIN AND NORTHERN IRELAND

## Abstract

**Background:**

Total hip and knee arthroplasties significantly improve the quality of life for patients with severe osteoarthritis. However, some patients experience complications that require follow-up care. Amid rising demand for these arthroplasties, debates have emerged around the value of routinely scheduled follow-ups (RFUs). This qualitative study, nested within a hybrid effectiveness de-implementation trial that assesses quantitative differences between RFU and check-ups on-demand (COD), will explore and compare the patients’ and health care professionals’ (HCPs) experiences with, and perceptions about, RFU and COD at 1 and 10 years after total hip and knee arthroplasty.

**Materials and methods:**

First, a pre-study reference panel will be organised to prepare a focus groups topics guide. Subsequently, 2 methods of data collection will be used: 8 focus groups with total of 80 participants from the main trial and 10 in-depth interviews with different HCPs. Thematic analysis using a deductive approach will be performed on anonymised transcripts to identify key themes. For this, the comparative case study framework and the Theoretical Framework of Acceptability will be used. Findings will be used to inform the transition from RFU to COD if deemed acceptable by patients and HCPs.

## Introduction

Total hip arthroplasty (THA) and total knee arthroplasty (TKA) are effective and safe surgeries that improve quality of life for patients with severe osteoarthritis [1,2]. Radiographic follow-up studies have shown that between 0–3% of patients develop post-operative complications requiring further treatment or revision surgery within 6 weeks to 1 year after surgery [3,4]. These include dislocation, infection, osteolysis or periprosthetic fracture (e.g., [5–10]). While these complications occur relatively infrequently, their early identification is crucial [11] and this explains why most patients after THA and TKA are still scheduled for routine follow-ups (RFUs) in hospital. Despite expert support for these radiographs and follow-ups [12] based on low-level evidence information and guideline recommendations [13–17], there is little consensus on their overall value, optimal frequency, and timing, leading to considerable variation in clinical practice [18,19].

With rising total joint arthroplasty demand due to ageing [20,21] and increasing rates of obesity [22,23], hospitals face pressure work more efficiently to meet waiting list targets while maintaining budgets [19]. This has sparked debate among orthopaedic HCPs about the need for, and usefulness of, several of the scheduled RFUs [10,19,24,25]. There is mainly discussion around de-implementing follow-ups for patients with well-established prostheses [26]. After all, symptomatic complications beyond one year after the index procedure are infrequent [27,28], most symptomatic patients do not require surgical intervention and surgeons can effectively identify patients likely to experience prosthesis failure for follow-up [29]. Additionally, most complications are not even detected during RFUs, but rather during unplanned hospital visits [25,30,31]. Repeated, uneventful follow-up visit can lead to frustration for both patients and orthopaedic HCPs [19]. Refraining from these can save costs [32–34], reduce CO_2_ emissions [33,35] and may reduce hospital wait times and wait lists.

While both patients and HCPs acknowledge the benefits of reducing in-person RFUs in terms of patient convenience and resource allocation, concerns remain about missing the opportunity to detect harmful issues with arthroplasties before irrevocable damage occurs [18,19]. Disinvestment in follow-ups may also weaken the human connection central to clinical care, contributing to both patient and HCP dissatisfaction [19,34,36] even though patients have also indicated they found reduced or alternative follow-up methods satisfying (e.g., [32,33,37,38]). Despite ongoing debate and a lack of high-quality evidence, many hospitals have already decided to reduce or even eliminate follow-ups [10,19,39].

Before RFUs after THA and TKA are de-implemented, it is crucial to understand what patients and HCPs find important in this regard for reasons of safety, quality of care and respecting patient preferences. Qualitative research is well-suited for exploring such perceptions [40]. A previous qualitative interview sub-study explored views of professional leads and service managers of orthopaedic hospital departments about implementing new care pathways such as de-implementing follow-ups for patients after THA and TKA [19,41]. Although interviewees felt that ‘traditional’ follow-ups frequently offered little benefit to the patient and suggested that a more meaningful form of follow-up should be adopted, the views of patients themselves nor the HCPs conducting these visits were explored. Experiences and perceptions of both patients after THA and TKA and two of their clinicians were evaluated in a transition from a traditional follow-up clinic to a virtual clinic pathway [34]. This transition was well accepted overall, but both groups agreed that patients with concerns or symptoms should have easy access to face to-face review, regardless of when these concerns or symptoms occur. To our knowledge, research on transitioning from a routinely scheduled follow-up regimen to a demand-driven follow-up regimen which allows patients and HCPs to seek follow-up only as needed, at their discretion, has yet to be conducted. This protocol paper outlines a qualitative study within a hybrid effectiveness de-implementation trial which will explore the experiences and perceptions of patients and HCPs regarding both RFU and check-ups on-demand (COD), and assess COD acceptability.

## Methods and materials

### Design and objectives

**Fig 1:**
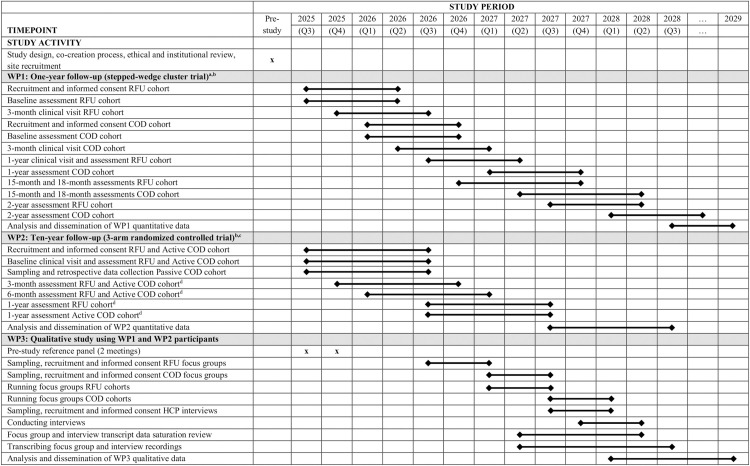
Adapted SPIRIT Schedule of Enrollment, Interventions, and Assessments summarising all 3 work packages of the nationwide hybrid effectiveness de-implementation trial. a This part of the study recruits patients scheduled for total hip or knee arthroplasty. A staggered crossover process is used whereby each of the 10 participating hospitals/sites – in a randomised order – sequentially closes recruitment for RFU and (after a two-month transitioning phase) initiates recruitment for COD. b During WP1 and WP2 assessments, outcomes include PROMIS physical function, healthcare consumption (clinical visits, radiographs), the number and type of complications and surgical interventions, health related quality of life, pain, satisfaction and healthcare costs. c This part of the study recruits patients who had total hip or knee arthroplasty 10 years ago. Only the RFU and Active COD cohorts will be assessed. For a Passive COD cohort, only retrospective registry data will be collected. d Participants are enrolled in WP2 approximately 10 years after undergoing total hip or knee arthroplasty. Therefore, the time points referred to as “3-month”, “6-month”, and “1-year” assessments should be understood as 10 years + 3 months, 10 years + 6 months, and 11 years after surgery. These labels reflect the time elapsed since enrolment in this follow-up phase, not since the original procedure. COD, check-up on demand; HCP, health care professional; RFU, regular follow-up; WP, Work Package.

This qualitative study is nested within a large nationwide hybrid effectiveness de-implementation trial (https://projecten.zonmw.nl/nl/project/standaardcontroles-na-een-totale-heup-knieprothese-verspilde-moeite-passende-zorg-haka) conducted in 10 non-academic hospitals in the Netherlands. A large group of stakeholders co-created this trial and secured a grant intended for studies aimed at investigating and improving the efficiency and cost-effectiveness of specialized medical care (https://www.zorgevaluatienederland.nl/evaluations/haka-1-year-fu and https://www.zorgevaluatienederland.nl/evaluations/haka-10-year-fu). The SPIRIT Schedule of Enrollment, Interventions, and Assessments (Fig 1) was adapted to summarise the 3 separate work packages of this trial. The qualitative work package (protocol version 1.0 of 14 February 2025) was granted a waiver of ethical approval on March 19, 2025 by the Leiden-Den Haag-Delft Medical Research Ethics Committee (reference number N25.014) and registered on 19 June 2025 (Clinicaltrials.gov ID: NCT07041528, https://clinicaltrials.gov/study/NCT07041528). The nationwide main trial is expected to start in July 2025, with the first qualitative work package activities commencing thereafter.

New patients planned for THA or TKA surgery (work package 1) and patients having had their THA or TKA index surgery 10 years ago (work package 2) will receive RFU or COD. The trial’s main work packages 1 and 2 will investigate the difference in effect between RFU and COD regimens on physical functioning, post-operative complications, quality of life, pain and healthcare consumption at selected time-points after the index surgery. Subsequently, this qualitative study (work package 3) will explore and compare the patients’ and HCPs’ experiences with, and perceptions about, the two different ‘cases’ of RFU and COD using the comparative case study framework [42]. In particular, the concurrent acceptability of the COD regimes will be explored using selected component constructs of the Theoretical Framework of Acceptability [43]. Two methods of data collection will be used: focus groups with participants and in-depth interviews with HCPs. Prior to conducting these, a pre-study reference panel will be organised to prepare the focus groups topics guide.

### Consent

All participants will first provide written informed consent to take part in either the main trial’s 1-year or 10-year follow-up study. Within the consent form, participants can indicate their willingness to participate in a focus group. Participants selected for focus group participation will additionally be asked to sign a separate informed consent form. Reference panel members will also be asked to provide verbal consent to be consulted, and HCPs will be asked to provide written informed consent to be interviewed.

### Patient and public involvement: Pre-study reference panel

Before any focus groups are conducted, we will recruit 10 people [44] with lived experience of THA and TKA surgery through network contacts within non-profit patient advocacy organizations for people with rheumatic conditions (ReumaZorg Nederland) and osteoarthritis (Poly-Artrose Lotgenoten Vereniging). In the second half of 2025, these people will be invited to share their experiences with post-operative follow-ups and will be asked to assist the researchers in formulating appropriate, actionable, clear, precise and relevant open-ended questions for the focus groups.

During a 2-hour meeting, researchers will first frame the aim and procedures of the reference panel [44]. After the panel members have been asked about their background and about their experiences with THA or TKA surgery and hospital follow-ups, they will receive evidence-based information about the main drivers of acceptability of, and patient satisfaction with, clinical services. Subsequently, the panel members’ feedback will be sought on a tentative focus group topics guide prepared by the researchers. During this process, panel members will be asked if any additional themes need to be explored, and to formulate open-ended questions for these. During a second 1-hour meeting, and until group consensus is reached, the researchers will seek the panel members’ feedback on a refined interview topics guide, and ensure that all questions are easy, clear, and understandable.

### Focus groups

A purposive sample of 80 participants who completed the main trial’s 1-year or 10-year follow-up studies will be approached and recruited for the post-intervention focus groups. Comparing these participants’ experiences and perceived satisfaction with their respective follow-up regimens will help determine if de-implementing RFU and adopting COD is acceptable.

Participation in the focus group is only possible for participants with a good command of the Dutch language. Participant sampling will further be based on variation in gender, age, traveling distance to the hospital and the American Society of Anesthesiologists (ASA) physical status classification. We will also strive to include participants who have had revision arthroplasty during participation to ensure that their perspectives are also captured for reasons of data adequacy [45,46]. A total of 8 focus groups of approximately 60–90 minutes will be conducted between January 2027 and April 2028, each consisting of 10 participants. Four focus groups will be organised for participants in the 1-year follow-up study, covering the following categories: THA with RFU, THA with COD, TKA with RFU, and TKA with COD. Similarly, 4 focus groups will be organised for participants in the 10-year follow-up study. All focus groups will be attended by at least 1 experienced qualitative researcher and 1 junior researcher, with each either moderating the discussions or taking field notes. All focus group venues will be easily accessible in terms of parking, physical access and public transport. Focus group participants will receive a 25-euro reimbursement for their travel and parking expenses.

A mix of semi-structured open-ended focus group questions will primarily be structured around themes that have emerged during previous research aimed at evaluating factors that influence patient satisfaction in general, and with medical consultation in particular. These studies have shown that interpersonal factors dominate perspectives of patient satisfaction [47]. An important theme in this regard is communication between caregivers and patients [47–50], for example communication about medical information that patients need or receive during a follow-up visit. Patients’ perceptions about being listened to, being heard or being helped by the provider can be captured under the theme of empathy [47,51,52] while trust has also been identified as a primary driver of patient satisfaction with clinical services [47,53]. Patients’ expectations also influence their degree of satisfaction [47,49,54]. Clinic environmental factors can also play a role such as the clinics’ responsiveness and easy accessibility [48] when patients request an appointment or the amount of clinical contact time [47,50,55,56] available to ask questions and address the patients’ concerns. Participants will be asked about perceived health risks (‘safety’) with, barriers and facilitators of, likes and dislikes about, and recommendations for alternative models of RFU and COD. To understand if the follow-up regimens are acceptable to patients, some additional questions will also be structured around the seven component constructs of the Theoretical Framework of Acceptability (v2) [43,57]. One question will ask participants about their fear-avoidance beliefs to help gauge the effectiveness of RFU and COD in terms of patient concerns or reassurance. Another question will explore whether—and why—patients’ satisfaction with their prosthesis is related to their preference for scheduled RFUs or COD. Additional questions may arise based on input from the pre-study reference panel.

### In-depth interviews

From the broader pool of HCPs working in or with the participating hospitals—such as orthopaedic surgeons, junior orthopaedic doctors, physician assistants, referring general practitioners, and physiotherapists—who conducted RFU or COD visits, or treated patients during the main trial, a purposive sample of 10 HCPs will be selected for post-intervention in-depth interviews. Exploring these HCPs’ experiences and perceived satisfaction with the 2 different follow-up regimens will assist in determining whether they feel it is acceptable to de-implement RFU. Sampling for the interviews will be based on variation in role, gender and working experience (in years). Only HCPs who have cared for at least 3 participants from the RFU or COD cohorts will be eligible to be interviewed. Thirty-minute interviews will take place between October 2027 and July 2028, either at the HCP’s own hospital or by phone. For the first 3 interviews, a senior researcher with qualitative research expertise will supervise the junior researcher for training purposes. Subsequently, the junior researcher will conduct the remaining interviews independently.

A mix of semi-structured open-ended interview questions will be structured around themes deemed relevant to HCPs who perform the follow-ups of patients after THA and TKA. In theory, de-implementation of RFU in favor of COD can hinder detection of prosthesis failure and increase the risk of missing (signs of) complications. As such, the theme of patient safety will primarily be probed. De-implementation of RFU will arguably also change the HCPs’ daily work practice. Apart from issues of managing the patients’ expectations [54] during COD follow-ups, this may also instill feelings of fear of losing touch [58] with patients. Finally, HCPs will also be asked about barriers and facilitators of, and likes and dislikes about RFU and COD, as well as about potential alternative models follow-up after THA or TKA.

### Qualitative data management plan

All focus group sessions with participants and in-depth interviews with HCPs will be recorded. Recordings will be fully transcribed verbatim. Transcripts will be coded before analysis. During the study, a researcher experienced in qualitative research will review the focus group and interview transcripts to determine if data saturation has been reached, or if additional focus groups or interviews are needed until no new information emerges.

Thematic analysis will be conducted using a deductive approach. First, 2 researchers will independently read through the transcripts to familiarise themselves with the data. They will then collaboratively organise codes under main (candidate) themes and subthemes through a stepwise categorisation process. This process will first be applied separately to the RFU and COD focus group transcripts. Upon completion, a cross-case comparison will be made to identify similarities and differences in perceptions of acceptability across the different cases, with coding based on the component constructs of the TFA framework. A similar process will be repeated for the interviews with the HCPs, but here a report detailing the specific descriptions of themes will be provided to the interviewees for member checking. This will ensure accuracy and validity of the findings [40]. In the final report, representative quotes will be included to support the identified themes. Focus group quotes will be labelled to indicate the participants’ gender (F, M), surgical joint (THA, TKA), and in which cohort they participated (1-year or 10-year follow-up study, RFU or COD). Quotes of HCPs will be labelled to indicate their gender and professional role. Depending on the findings, an overarching theme will be formulated to summarise, or a concluding framework will be constructed to conceptualise, the experiences and perceptions of patients and HCPs that are important during the transition to a new follow-up regimen. All methods will be reported according to the Consolidated Criteria for Reporting Qualitative Research (COREQ) [59].

## Discussion

The demand for THA and TKA continues to grow worldwide [20,21]. As a consequence, healthcare systems face increasing pressure to balance quality of THA and TKA follow-up care with economic sustainability [19]. Previous research has shown that complications after primary THA and TKA within and beyond the first year after surgery are relatively rare [27,28]. There is also limited and low-quality evidence supporting the necessity of routinely scheduled follow-ups, particularly for patients with well-functioning prostheses [26] and without any symptoms. Taken together, these findings suggest that RFUs could be de-implemented altogether, but concerns of patients and HCPs remain about the potential for missed diagnoses and patient safety [18,19].

A proposed nationwide hybrid effectiveness de-implementation trial, which has the potential to provide high-level evidence on differences between RFU and COD, also presents an opportunity to explore and compare patients’ and HCPs’ experiences with and perceptions of RFU and COD. The qualitative exploration described in this protocol paper may provide valuable insights into the acceptability and potential consequences of transitioning from RFU to COD, thereby contributing to the ongoing discussion surrounding the optimisation of post-operative follow-up care after THA and TKA. The perspectives of patients are particularly crucial, as their perceptions of safety, convenience, and satisfaction play a significant role in determining the acceptability of alternative follow-up models. Previous research has suggested that patients generally support reduced follow-ups if they retain the ability to seek medical attention when needed [32]. Our findings may help elucidate whether this view extends to a demand-driven approach. From the perspective of HCPs, COD may present challenges related to maintaining clinical oversight and managing patient expectations. Our study will explore how HCPs perceive the feasibility and impact of COD, including potential barriers to implementation and strategies to mitigate risks. Concerns about losing touch with patients and the implications for long-term prosthesis survival will also be explored, adding depth to the current discussion on follow-up de-implementation. Ultimately, the findings of this study may help determine whether replacing scheduled RFUs with a demand-driven model can be implemented effectively and safely.

### Strengths and limitations

A key strength of this study is its integration within a larger hybrid effectiveness de-implementation trial, which allows for supplementing quantitative outcomes with qualitative insights. This combined approach will provide a comprehensive evaluation of the effectiveness, safety, and acceptability of transitioning from a RFU to a COD regimen. This study will also collect qualitative data from 2 of the most important stakeholders of follow-up visits after THA and TKA: patients and their health care professionals. Triangulating the findings of these data sources enables rich and meaningful understanding of how RFU and COD are perceived. Finally, the use of the comparative case study framework and the Theoretical Framework of Acceptability will ensure that findings are interpreted within a structured, evidence-based context.

Some limitations of this study also deserve note. Participants in this qualitative study are drawn from Dutch hospitals only, which may limit the generalisability of findings to other countries with different healthcare systems and institutions with different follow-up practices. Patients who opt into this qualitative part of the main trial may already have stronger opinions about RFU or COD than the average patient, leading to an overrepresentation of extreme views. Finally, since the focus groups will take place approximately 9 months after the final clinical visit for participants who had COD, recall bias also remains a possibility.

### Dissemination of findings

The findings of this study will be disseminated through peer-reviewed international journal publications and conference presentations. In addition, results will be disseminated through the co-creating patient advocacy organisations ReumaZorg Nederland, Poly-Artrose Lotgenoten Vereniging and the Netherlands Orthopaedic Association (NOV). Supporting data—including an overview of all themes, original Dutch quotes from focus groups and interviews, and their English translations—will be compiled in a table and made openly available via an online data repository.

## Supporting information

S1 Standard Protocol Items Recommendations for Interventional Trials (SPIRIT, 2013) Checklist. (PDF)

S1 File English translation of the original Dutch study protocol (version 1.0 of 14-02-2025) submitted to Leiden-Den Haag-Delft Medical Research Ethics Committee (reference number N25.014). (PDF)
