# Supplementary material for: How do patients and health care professionals perceive de-implementation of routine follow-ups after total hip or knee arthroplasty? Protocol for a nested qualitative study within a hybrid effectiveness de-implementation trial
Source: PLoS One. 2025 Aug 28;20(8):e0330652. doi: 10.1371/journal.pone.0330652 (PMC12393720; doi:10.1371/journal.pone.0330652)
Supplement: S1 File — (PDF) [file pone.0330652.s002.pdf]

STUDY INFORMATION

Title of the Study
How do patients and health care professionals perceive de-implementation of routine follow-ups after total hip and knee arthroplasty? Protocol for a nested QUALItative study within a hybrid de-implementation trial (HAKA trial).

Acronym / Short Study Title
HAKA-QUALI

Date / Version
14-FEB-2025 / Version 1.0

Type of Study
☐ Retrospective study (file/status review)
☐ (Partly) prospective study
☒ Other, namely: exploratory qualitative study

Sponsor / Executor

The sponsor/executor is the institution (hospital, company, etc.) that commissioned the organization and/or execution of the study: **Tergooi MC**

Research Team Members

Name	Role	Department & Institution
Ronald Verhagen (orthopedic surgeon)	Principal Investigator	Orthopedics, Tergooi MC
Dominique Baas	Coordinating Researcher	Research coordinator, Orthopedics, Tergooi MC
Lidy Roubos, PhD student	PhD Candidate	OLVG
Lex de Jong	Local Researcher	Research coordinator, Orthopedics, Rijnstate
Job van Susante	Local Principal Investigator	Orthopedic surgeon, Orthopedics, Rijnstate
Ariena Rasker	Local Researcher	Researcher, Orthopedics, OLVG
Rudolf Poolman	Local Principal Investigator	Orthopedic surgeon, Orthopedics, OLVG
Jantsje Pasma	Local Researcher	Researcher, Orthopedics, RHOC
Marijn Rutgers	Local Principal Investigator	Orthopedic surgeon, Orthopedics, RHOC
Nienke Willigenburg	Local Researcher	Senior researcher, Orthopedics, OLVG

Submitter (METC LDD submission contact)

Name: Dr. D.C. Baas (Tergooi MC)
Phone: [redacted]
Email: [redacted]

Local Principal Investigator
Name: Ronald Verhagen (Tergooi MC)
Phone: [redacted]
Email: [redacted]

Conducted in the Context of

☐ General scientific research (e.g., within institutional research line)

☒ PhD research

☐ Scientific internship / bachelor’s or master’s thesis. Name of institute:

☒ Other, namely:
ZonMw subsidy "Appropriate Care Evaluation" – 2023 Round, Questions from knowledge agendas of medical specialist care.

TABLE OF CONTENTS

1. Summary
2. Introduction
3. Research Question / Study Objective
4. Methods
    - 3.1 Single or Multicenter Study
    - 3.2 Study Design
    - 3.3 Procedure and Intervention (if applicable)
    - 3.4 Study Duration
    - 3.5 Recruitment and Selection of Participants
    - 3.6 Data Collection: Variables and Measurement Methods
    - 3.7 Data Analysis
5. Ethical Considerations
    - 4.1 Non-WMO Statement
    - 4.2 Participant Burden and Compensation
    - 4.3 Informed Consent
6. Data Management & Privacy
    - 5.1 Data Storage, Security and Access During the Study
    - 5.2 Data Processing
    - 5.3 Data Sharing
    - 5.4 Data Retention Period
7. Valorization and Publication
    - 6.1 Valorization
    - 6.2 Publication
8. References

SUMMARY

Total hip and knee arthroplasty (THA and TKA) are highly successful procedures that significantly improve the quality of life for people with severe osteoarthritis. However, a small percentage of patients experience postoperative complications, for which early detection is crucial. Therefore, patients are routinely scheduled for follow-up appointments (RFUs) in the months and years after surgery.

Due to population aging and increasing healthcare demands, waiting lists and costs for THA and TKA are rising, while scientific evidence supporting the effectiveness of repeated hospital check-ups is limited. This has led to debate on the necessity of multiple RFUs per patient and whether a transition to follow-up initiated only on patient or provider request — check-up on demand (COD) — could be equally effective, safe, and acceptable.

The HAKA trial consists of three studies: a 1-year and a 10-year follow-up study, and a qualitative study (HAKA-QUALI — this protocol concerns the latter). The 1- and 10-year studies examine differences between RFU and COD in physical functioning, complications, quality of life, pain, and healthcare usage.

While previous research has shown that healthcare professionals recognize the benefits of reducing RFUs, there are concerns about missing complications and the loss of patient-provider interaction. Yet, many patients report satisfaction with fewer hospital visits or alternative follow-up formats.

In the HAKA-QUALI study, we explore patient and provider experiences and perceptions of RFU and COD. We will conduct eight focus groups with patients from the HAKA trial and interview ten healthcare professionals. Their experiences and views will be analyzed and categorized using existing theoretical frameworks. Based on these findings, we expect to make recommendations on the acceptability of eliminating routine check-ups for both patients and providers.

1. INTRODUCTION

Total hip and knee arthroplasty (THA and TKA) are highly successful surgical procedures that can significantly improve the quality of life for people with severe joint osteoarthritis [1, 2]. Although both surgeries are effective and safe, studies show that follow-up (FU) within one year after surgery results in complications in 0–4.6% of patients, for which only a small proportion require additional treatment or revision surgery [3, 4]. Examples include dislocation, infection, osteolysis, prosthesis loosening, or periprosthetic fracture [5–10].

Since early detection of these complications is important [11], most patients are called back multiple times for routine hospital follow-up. Despite expert support for these routine follow-ups (RFUs) [12], the available scientific evidence is weak [13–17], and there is little consensus on the optimal frequency and timing of RFUs, leading to significant variation in practice [18, 19].

The rising demand for THAs and TKAs, due to an aging population and increased obesity rates [19, 20], is putting pressure on waitlists and healthcare budgets [19]. This has sparked debate among orthopedic professionals about the necessity and utility of RFUs [11, 19, 22,

23]. Furthermore, surgeons are capable of identifying patients at greater risk of prosthesis failure [24], who can receive extra RFUs if needed. Additionally, most complications are not detected during scheduled RFUs but during unscheduled visits [23, 25, 26].

Conducting many RFUs — which rarely reveal issues — can cause dissatisfaction among patients and providers [24] due to travel costs [27–29], CO_2 emissions [28, 30], and prolonged wait times. While providers acknowledge the benefits of reducing RFUs, they also express concern about missing complications [18, 19]. Reducing RFUs may also negatively affect the vital human interaction between patients and providers — a loss that can lead to dissatisfaction [19, 29, 31].

Nevertheless, patients report that fewer hospital visits or alternative RFU models are acceptable [27, 28, 32, 33]. Despite ongoing debate and limited evidence, many hospitals have already reduced or redesigned RFU programs [10, 19].

If de-implementation of RFU is considered after THA and TKA, it is crucial to understand what matters to patients and providers. Qualitative research is a proven method to study such perceptions [34]. To date, no extensive qualitative research has examined the transition from RFU to patient- or provider-initiated follow-up only (check-up on demand, COD).

This protocol describes the qualitative HAKA-QUALI study, which will explore the experiences and perceptions of patients and providers regarding both RFU and COD, as part of a larger ZonMw-funded project (HAKA trial), briefly summarized below:

- The **HAKA trial** compares RFU vs. COD on physical function, complications, quality of life, pain, and care use at 1 and 10 years post-surgery.
  - The **1-year FU study** (not covered by this protocol) is a multicenter stepped-wedge cluster trial. Each site starts with RFU (control) and later switches to COD (intervention), which becomes the local standard. Half the participants receive RFU (3-month and 1-year check-ups); the other half receive COD (only a 3-month check-up plus guidance on when and how to request follow-up).
  - The **10-year FU study** (also not covered here) uses a cross-sectional randomized design with 3 groups: RFU, active COD (ACOD), and passive COD (PCOD). ACOD participants are contacted, informed, and asked to complete surveys; PCOD participants are not contacted — only anonymous EHR data is used.

---

2. RESEARCH QUESTION / OBJECTIVE

This qualitative HAKA-QUALI study explores the experiences and perceptions of patients and healthcare professionals regarding RFU and COD. The main aim is to assess whether the new COD model is acceptable and safe from both perspectives.

---

3. METHODS

3.1 Single or Multicenter Study

☐ Single-center
☐ Multicenter

Participating Centers and Local PIs:

- Tergooi MC (Dr. R. Verhagen, sponsor)
- OLVG (Prof. Dr. R. Poolman)
- RHOC (Dr. M. Rutgers)

3.2 Study Design

HAKA-QUALI is a qualitative study embedded in a national hybrid effectiveness/(de-)implementation trial: the HAKA trial.

Two data collection methods will be used:

- Single-session **focus groups** with patients
- Single **one-on-one interviews** with healthcare professionals

Patient participants will be recruited from the 1- and 10-year FU studies within HAKA. Prior to data collection, 10 patient experts will be invited to form a **reference panel** (details below).

3.3 Procedure and Intervention (if applicable)

Reference Panel

Ten patient experts (THA/TKA recipients) will help design the focus group questions:

- **Meeting 1:** Feedback on draft questions and topics.
- **Meeting 2:** Review updated questions for neutrality, clarity, simplicity, and precision. Suggest additions if needed.

Note: The reference panel is not part of the formal study but serves to improve quality and relevance.

Focus Groups

Patients from 3 centers (OLVG, Tergooi MC, RHOC) in the HAKA trial will be selectively sampled (see 3.5.1). They will join one 60–90-minute focus group at the hospital where they had surgery.

Total of **8 focus groups**:

- **1-year FU:** 4 groups (1 per joint x 2 follow-up types: THA-RFU, THA-COD, TKA-RFU, TKA-COD)
- **10-year FU:** 4 groups (same structure as above)

Focus group questions will explore safety perceptions, barriers/facilitators, pros/cons of RFU vs. COD, and perceived acceptability (using the Theoretical Framework of Acceptability — TFA) [36, 37].

Provider Interviews

Ten orthopedic care providers (residents, surgeons, PTs) from the same 3 centers will be recruited for a single 30-minute one-on-one interview.

Topics will include:

- Patient safety
- How RFU/COD affect daily practice
- Perceived pros/cons
- All questions will be open-ended and clearly phrased

3.4 Duration of the Study

- Reference panel: 12 months
- Data collection (focus groups + interviews): 21 months
- Data analysis: 6 months

3.5 Recruitment and Selection of Participants

Screening / Selection

Reference Panel
Ten patient experts will be recruited via representatives of the patient associations:

- ReumaZorg Nederland
- Poly-Artrose Lotgenotenvereniging (P-AL)

These representatives are members of the overarching HAKA trial research team (see 4.3).

Focus Groups
Participants will be selected from the 1- and 10-year FU study groups of HAKA at OLVG, Tergooi MC, and RHOC.
Participants who gave permission to be contacted will be selected for variation in:

- Gender
- Age
- Travel distance

- Physical health (ASA classification)

Interviews
Healthcare providers from the same 3 centers will be recruited by local researchers, aiming for variation in:

- Role
- Gender
- Years of experience

Study Population

- **1-year FU study**: Patients scheduled for primary THA or TKA
- **10-year FU study**: Patients who had a THA or TKA 10 years ago
- **Healthcare providers**: Orthopedics staff at OLVG, Tergooi MC, or RHOC

Inclusion Criteria

Patients:

- Diagnosis of osteoarthritis
- Scheduled for primary THA or TKA
- Aged 50 or older
- Sufficient command of Dutch

Providers:

- Experience with ≥3 RFU/COD follow-up cases

Exclusion Criteria

- Other indication than osteoarthritis
- Scheduled for revision surgery (except conversions, e.g. from hemiarthroplasty to THA)

Sample Size

- 10 patient experts for the reference panel
- 80 patients for focus groups
- 10 providers for interviews

3.6 Data Collection: Variables and Methods

Primary Outcome (dependent variable):
Perceptions (experiences, opinions) about the follow-up model used in the HAKA trial. Topics include:

- Acceptability
- Satisfaction
- Safety concerns
- Barriers and facilitators
- Pros and cons
- Recommendations for future follow-up

Data Frameworks Used:

- *Comparative Case Study Framework* [35]
- *Theoretical Framework of Acceptability (TFA)* [37, 38]

Standardization
Focus group questions are based on the TFA and prior literature on patient satisfaction with medical consultations. These will be refined with input from the reference panel.

Interview questions for providers focus on:

- Patient safety
- Impact of RFU/COD on their daily practice
- Clarity and neutrality of language ensured

3.7 Data Analysis

Inspection
After 8 focus groups and 10 interviews, a qualitative researcher will assess transcript saturation. If needed, additional sessions will be conducted.

Thematic Analysis (deductive approach):

- Two researchers independently review anonymized transcripts
- Coding under main themes and subthemes
- Separate analyses for RFU and COD groups
- Based on TFA
- Member-checking: summaries returned to participants for verification
- Use of NVivo, Word, and Excel
- Reporting per COREQ guidelines [39]

4. ETHICAL CONSIDERATIONS

4.1 Non-WMO Declaration

This study is being submitted for a non-WMO statement.

4.2 Burden and Compensation

Reference Panel:

- Two meetings (2h and 1h)
- €25 per meeting for travel and parking
- Catering provided

Focus Groups:

- One session (60–90 minutes)
- €25 compensation
- Held at the hospital of surgery
- Catering provided

Interviews:

- 30-minute one-on-one session
- Conducted by phone or on-site
- No travel, so no compensation

4.3 Informed Consent

Required?

☒ Yes
☐ No

Procedure – Option A:

Reference Panel:

- Invited by patient association representatives
- Informed via personal contact (not via formal patient info form)

Focus Groups:

- Consent to contact given during HAKA trial
- Phone call to confirm interest and send Patient Info + Consent Form (PIF + ICF)
- After 5-day reflection, follow-up call for confirmation
- Return of signed consent forms via prepaid envelope

Interviews:

- Recruited via local staff
- Sent PIF + ICF
- After 5-day reflection period, phone follow-up for questions and final consent

---

5. DATA MANAGEMENT & PRIVACY

5.1 Data Storage, Security & Access

Paper Data:

- Stored in Investigator Site File (ISF) at Tergooi MC
- Locked cabinet in locked room
- Accessible only by HAKA study team (2+ members)

Digital Data:

- Stored in Excel on SharePoint (Tergooi MC)
- Access: HAKA study team
- Secured by password and restricted folder access

5.2 Data Processing

Anonymization and Coding:

☒ Anonymized

☒ Coded

- Focus group and interview audio recorded (MP3)
- Transcripts created by an external party
- Audio deleted after transcription
- Quotes coded by gender, study group (1- or 10-year, RFU or COD), and joint (hip/knee)
- Identifying data (names, places) anonymized

Code Key:

- Stored in SharePoint (Tergooi MC), password-protected

- Access restricted to study team

Data Extracted by:

- PhD student (under supervising physician)

5.3 Data Sharing

☒ Yes, anonymized
☒ Yes, coded
☐ No

Shared with:

- Dutch researchers: PhD student, RHOC, OLVG, Rijnstate

5.4 Data Retention

- Audio files deleted after transcription
- Anonymized transcripts stored for 15 years

---

6. VALORIZATION & PUBLICATION

6.1 Valorization

This study will provide in-depth insight into how patients and providers perceive RFU and COD after THA or TKA. Focus group findings will inform:

- Acceptability
- Satisfaction
- Barriers/facilitators
- Safety concerns
- Recommendations

Providers will reflect on:

- Clinical implications
- Effects on safety and workflow

Findings may lead to better-designed follow-up protocols.

6.2 Publication

Results will be submitted to an international peer-reviewed journal (to be determined), and presented at:

- Dutch Orthopedic Association (NOV) Annual Congress
- A future international orthopedic conference

---

7. REFERENCES

[Includes references 1–39 as listed in the original Dutch document.]

1. Learmonth, I.D., C. Young, and C. Rorabeck, The operation of the century: total hip replacement. Lancet, 2007. 370(9597): p. 1508-19.

2. Price, A.J., et al., Knee replacement. Lancet, 2018. 392(10158): p. 1672-1682.

3. Hart, A.A., et al., Routine Radiographs After Total Joint Arthroplasty: Is There Clinical Value? J Arthroplasty, 2021. 36(7): p. 2431-2434.

4. Christensen, M. and K. Folkmar. No clinical value of post-operative routine X-ray following uncomplicated cementless primary total hip arthroplasty. Dan Med J, 2013. 60(4): p. A4613

5. Dargel, J., et al., Dislocation following total hip replacement. Dtsch Arztebl Int, 2014. 111(51-52): p. 884-90.

6. Blom, A.W., et al., Infection after total joint replacement of the hip and knee: research programme including the INFORM RCT, in Programme Grants for Applied Research. 2022: Southampton (UK).

7. Lindeque, B., et al., Infection after primary total hip arthroplasty. Orthopedics, 2014. 37(4): p. 257-65.

8. Schwartz, A.M., et al., Projections and Epidemiology of Revision Hip and Knee Arthroplasty in the United States to 2030. J Arthroplasty, 2020. 35(6S): p. S79-S85.

9. Kunutsor, S.K., et al., Risk factors for dislocation after primary total hip replacement: a systematic review and meta-analysis of 125 studies involving approximately five million hip replacements. Lancet Rheumatol, 2019. 1(2): p. e111-e121.

10. Cassidy, R.S., O.h. S, and D.E. Beverland, Guidelines for the follow-up of total hip arthroplasty: do they need to be revised? Bone Joint J, 2019. 101-B(5): p. 536-539.

11. Lovelock, T.M. and N.S. Broughton, Follow-up after arthroplasty of the hip and knee : are we over-servicing or under-caring? Bone Joint J, 2018. 100-B(1): p. 6-10.

12. Smith, L.K., E. Dures, and A.D. Beswick, Systematic review of the clinical effectiveness for long-term follow-up of total hip arthroplasty. Orthop Res Rev, 2019. 11: p. 69-78.

13. National Institute for Health and Care Excellence (NICE), Joint replacement (primary): hip, knee and shoulder (NICE guideline NG157). 2020.

14. American Academy of Orthopaedic Surgeons, American Academy of Orthopaedic Surgeons Surgical Management of Osteoarthritis of the Knee Evidence-Based Clinical Practice Guideline. 2022.

15. Broughton, N., et al., Arthroplasty Society of Australia guidelines for long term follow-up of joint replacement patients. 2019.

16. Federatie Medisch Specialisten, Total hip prosthesis (THP). 2019.

17. Federatie Medisch Specialisten, Richtlijn Federatie Medisch Specialisten Totale Knieprothese (TKP). 2021.

18. Loppini, M., et al., Large variation in timing of follow-up visits after hip replacement: a review of the literature. EFORT Open Rev, 2022. 7(3): p. 200-205.

19. Kingsbury, S.R., et al., Safety of disinvestment in mid- to late-term follow-up post primary hip and knee replacement: the UK SAFE evidence synthesis and recommendations. 2022: Southampton (UK).

20. George, J., et al., Obesity Epidemic: Is Its Impact on Total Joint Arthroplasty Underestimated? An Analysis of National Trends. Clin Orthop Relat Res, 2017. 475(7): p. 1798-1806.

21. Chen, L., et al., The burden of end-stage osteoarthritis in Australia: a population-based study on the incidence of total knee replacement attributable to overweight/obesity. Osteoarthritis Cartilage, 2022. 30(9): p. 1254-1262.

22. Smith, L.K., A survey of the current state of hip arthroplasty surveillance in the United Kingdom. Musculoskeletal Care, 2014. 12(4): p. 232-8.

23. Hacking, C., et al., Is there a need for routine follow-up after primary total hip arthroplasty? ANZ J Surg, 2010. 80(10): p. 737-40.

24. Kingsbury, S.R., et al., Mid- to late-term follow-up of primary hip and knee arthroplasty: the UK SAFE evidence-based recommendations. Bone Jt Open, 2023. 4(2): p. 72-78.

25. No authors listed. Orthopaedic Data Evaluation Panel (ODEP) Rating System. 2024 24-09-2024]; Available from: https://www.odep.org.uk/.

26. Pinedo-Villanueva, R., et al., Association between outpatient follow-up and incidence of revision after knee and hip replacements: a population-based cohort study. BMC Musculoskelet Disord, 2023. 24(1): p. 106.

27. Schmitz, P.P., et al., The (un)necessity of regular postoperative follow-up of hemiarthroplasty for femoral neck fractures. Injury, 2021. 52(10): p. 2997-3001.

28. Kingsbury, S.R., et al., A comparative study of patients presenting for planned and unplanned revision hip or knee arthroplasty. Bone Joint J, 2022. 104-B(1): p. 59-67.

29. El Ashmawy, A.H., et al., Effectiveness, Patient Satisfaction, and Cost Reduction of Virtual Joint Replacement Clinic Follow-Up of Hip and Knee Arthroplasty. J Arthroplasty, 2021. 36(3): p. 816-822 e1.

30. Richards, J.D., M. Stoddart, and B. Bolland, Virtual Arthroplasty Follow-Up: Better for the Trust, Patients, and the Planet. Cureus, 2022. 14(11): p. e31978.

31. Parkes, R.J., et al., Is virtual clinic follow-up of hip and knee joint replacement acceptable to patients and clinicians? A sequential mixed methods evaluation. BMJ Open Qual, 2019. 8(1): p. e000502.

32. Woolen, S.A., et al., Radiology Environmental Impact: What Is Known and How Can We Improve? Acad Radiol, 2023. 30(4): p. 625-630.

33. Zulman, D.M., et al., Practices to Foster Physician Presence and Connection With Patients in the Clinical Encounter. JAMA, 2020. 323(1): p. 70-81.

34. Healy, P., et al., Virtual outpatient clinic as an alternative to an actual clinic visit after surgical discharge: a randomised controlled trial. BMJ Qual Saf, 2019. 28(1): p. 24-31.

35. Yin, R., Case Study Research and Applications. Design and Methods. 6th ed. 2018, Thousand Oaks, CA: SAGE Publications, Inc.

36. Sekhon, M., M. Cartwright, and J.J. Francis, Acceptability of health care interventions: A theoretical framework and proposed research agenda. Br J Health Psychol, 2018. 23(3): p. 519-531.

37. Sekhon, M., M. Cartwright, and J.J. Francis, Development of a theory-informed questionnaire to assess the acceptability of healthcare interventions. BMC Health Serv Res, 2022. 22(1): p. 279.

38. Sekhon, M., M. Cartwright, and J.J. Francis, Acceptability of healthcare interventions: an overview of reviews and development of a theoretical framework. BMC Health Serv Res, 2017. 17(1): p. 88.

39. Tong, A., P. Sainsbury, and J. Craig, Consolidated criteria for reporting qualitative research (COREQ): a 32-item checklist for interviews and focus groups. Int J Qual Health Care, 2007. 19(6): p. 349-57.
